# The relationship between severe maternal morbidity and psychological health symptoms at 6–8 weeks postpartum: a prospective cohort study in one English maternity unit

**DOI:** 10.1186/1471-2393-14-133

**Published:** 2014-04-07

**Authors:** Marie Furuta, Jane Sandall, Derek Cooper, Debra Bick

**Affiliations:** 1 Department of Human Health Sciences, Graduate School of Medicine, Kyoto University, 53 Shogoin Kawara-cho, Sakyo-ku, Kyoto City, Kyoto 606-8507, Japan; 2 School of Medicine, King’s College London, London, UK; 3 Florence Nightingale School of Nursing and Midwifery, King’s College London, London, UK

**Keywords:** Stress disorders, Post-traumatic, Pregnancy complications, Puerperal disorders, Postnatal care, Cohort studies

## Abstract

**Background:**

The incidence of severe maternal morbidity is increasing in high-income countries. However, little has been known about the impact on postnatal morbidity, particularly on psychological health outcomes. The objective of this study was to assess the relationship between severe maternal morbidity (ie. major obstetric haemorrhage, severe hypertensive disorders or intensive care unit/obstetric high dependency unit admission) and postnatal psychological health symptoms, focusing on post-traumatic stress disorder (PTSD) symptoms at 6–8 weeks postpartum.

**Method:**

A prospective cohort study was undertaken of women who gave birth over six months in 2010 in an inner city maternity unit in England. Primary outcomes were prevalence of PTSD symptoms namely: 1) intrusion and 2) avoidance as measured using the Impact of Event Scale at 6 – 8 weeks postpartum via a self-administered postal questionnaire. Secondary outcomes included probable depression. Data on incidence of severe maternal morbidity were extracted from maternity records. Multivariable logistic regression analysis examined the relationship between severe maternal morbidity and PTSD symptoms taking into account factors that might influence the relationship.

**Results:**

Of women eligible to participate (n=3509), 52% responded. Prevalence of a clinically significant level of intrusion and avoidance were 6.4% (n=114) and 8.4% (n=150) respectively. There was a higher risk of PTSD symptoms among women who experienced severe maternal morbidity compared with women who did not (adjusted OR = 2.11, 95%CI = 1.17-3.78 for intrusion; adjusted OR = 3.28, 95%CI = 2.01-5.36 for avoidance). Higher ratings of reported sense of control during labour/birth partially mediated the risk of PTSD symptoms. There were no statistically significant differences in the prevalence or severity of symptoms of depression.

**Conclusion:**

This is one of the largest studies to date of PTSD symptoms among women who had recently given birth. Findings showed that an experience of severe maternal morbidity was independently associated with symptoms of PTSD. Individually tailored care that increases women’s sense of control during labour may be a protective factor with further work required to promote effective interventions to prevent these symptoms. Findings have important implications for women’s health and the content and organisation of maternity services during and after the birth.

## Background

As maternal mortality has declined in the UK, severe maternal morbidity is increasingly referred to as a more useful indicator of the safety and quality of maternity care [1]. However, little is known about the impact of severe maternal morbidity on women’s postnatal health. Studies have shown that the level of intervention during labour and birth affects the risk of experiencing fear and anxiety [2]. The combination of experiencing a life-threatening complication and necessary medical interventions may culminate in maternal psychological and physical morbidity [3]. This may in turn ‘trigger’ post-traumatic stress disorder (PTSD) in the postnatal period [4-6].

PTSD is a condition that can develop after a person has experienced or witnessed a highly traumatic event. PTSD involves three symptom clusters: intrusive recollection, avoidance and hyperarousal [7]. The Diagnostic and Statistical Manual of Mental Disorders (DSM-IV) recognises that an individual’s perception of threat and their response to an event critically affects subsequent development of PTSD [7]. Earlier studies have shown that PTSD affects approximately 2% to 6% of women at around six weeks following even spontaneous childbirth (i.e., full-term pregnancy with healthy outcome) using a range of measures [8,9]. A recent systematic review [10] identified the possibility that women’s experiences of maternal morbidity could trigger PTSD symptoms indirectly through a third factor such as infant condition (e.g. prematurity, death) and a woman’s perceived loss of control during labour and birth. However, due to methodological limitations including small sample sizes and unclear definition of severe maternal morbidity, the relationship between severe maternal morbidity and PTSD and possible mechanisms underlying the relationship could not be fully explained, leading to an evidence gap to support timely and appropriate care.

PTSD during the postpartum period is an important public health issue because of the longer-term negative impact of maternal mental health problems on child development [11-13] including impaired mother-infant relationship [14,15], delayed intellectual development [16,17] and psychiatric disorder in children [18]. Studies showed intrusion symptoms (eg. flashback memory or re-experiencing a traumatic birth) may affect women’s ability to adapt to motherhood and their relationships with others [2]. The experience of avoidance symptoms during the postnatal period may also impair a woman’s ability to talk about and process the trauma, leading to social isolation [2], with potential implications for her decisions about infant feeding [19,20]. Long-term maternal morbidity, if not identified or appropriately managed at an early stage, could also increase use of health care services by women and their families [21,22]. We therefore assessed the impact of severe maternal morbidity on postnatal maternal health among women who had recently given birth, focusing particularly on post-traumatic stress disorder (PTSD) at 6–8 weeks when routine UK maternity care provision ends. Objectives included to: 1) identify the prevalence of postnatal PTSD symptoms and other psychological outcomes among women who gave birth, 2) compare the rates of PTSD symptoms and other psychological outcomes in women who had severe maternal morbidity and those who did not; and 3) examine the relationship between severe maternal morbidity and PTSD symptoms taking into account factors that might influence the relationship.

## Methods

We undertook a prospective cohort study with severe maternal morbidity as the exposure and PTSD symptoms and postnatal depression symptoms as outcomes. After reviewing the definitions of severe maternal morbidity used in population-based studies in the UK and other high income countries [1,23-26], we selected two approaches to define this: disease-based and management-based. Disease-based definitions included major obstetric haemorrhage and severe pre-eclampsia/eclampsia/HELLP syndrome (a syndrome involving **h**aemolysis, **e**levated **l**iver enzymes **l**ow **p**latelets). Management-based definitions included admission to intensive care unit (ICU) or obstetric high dependency unit (HDU). Since almost all maternal complications after giving birth would be managed in the HDU in the study site, there was a considerable overlap between the two groups. However, including HDU admission in the management based group allowed us to identify less frequent types of severe maternal morbidity that would not be included in the disease based group. Women who had at least one episode of severe maternal morbidity (i.e. major obstetric haemorrhage, severe pre-eclampsia/eclampsia, HELLP syndrome, or ICU/HDU admission) were defined as having experienced severe maternal morbidity, while the remaining women were considered not to have experienced this.

### Primary outcomes

Our primary outcome was the prevalence of PTSD symptoms measured by 1) intrusion and 2) avoidance at 6–8 weeks using the Impact of Event Scale (IES) [27]. The IES is validated in general populations and is one of the most widely used self-report scales to measure PTSD symptoms in postnatal populations [28,29]. The IES only measures two of the three PTSD symptoms (intrusion and avoidance but not hyperarousal). Earlier studies of PTSD symptoms in postnatal populations [30] suggested that predictors or contributing factors of intrusion and avoidance symptoms might not necessarily be the same. Therefore, in this study, intrusion and avoidance were examined separately as primary outcomes. Hyperarousal symptoms were not included because some symptoms (e.g., difficulty in falling or staying asleep, difficulty concentrating or irritability) are difficult to distinguish from a normal state in the postpartum period [31] and inclusion could be counterproductive.

The IES includes 15 items, measuring the frequency of symptoms of intrusion (seven items) and avoidance (eight items) during the past week. Women were asked to report how often during the previous week they had experienced symptoms of distress related to an event or experience during their labour, the birth of their baby, or immediately after the birth (within 24 hours) that made them feel anxious and frightened. The items were scored on a four point scale: not at all (=0), rarely (=1), sometimes (=3) or often (=5) [27]. Using a standard commonly used cut-off, scores of “20 or more” in subscales (intrusion subscale score of 20 or more, or avoidance subscale score of 20 or more) were defined as a clinically significant level of distress [32], p.722. Based on the current study, the internal consistency of intrusion and avoidance with Cronbach’s alpha was 0.87 and 0.86, respectively.

### Secondary outcomes

As individuals who suffer from PTSD may experience several symptoms at the same time or over a period of time, we included other indicators of PTSD symptoms as secondary outcomes—specifically, the prevalence of *either* intrusion *or* avoidance (a total score of 20 or more on *either* IES subscales) and the prevalence of *both* intrusion *and* avoidance (a total score of 20 or more on *both* subscales). Other secondary outcomes relevant to psychological health included continuous measures of symptoms of PTSD using IES scores and probable major depression based on a score of 13 or more on the Edinburgh Postnatal Depression Scale (EPDS) [33], with severity based on a high EPDS score. Studies in the UK have shown that using a threshold EPDS score of 12/13 in the sixth week postnatal, that sensitivity ranges from 68% to 95% and specificity from 78% to 96% compared to a diagnosis of major depression following psychiatric interview [33-35].

We also included routine and non-routine consultations with healthcare professionals, breastfeeding practice and general health as secondary outcomes, which will be reported elsewhere.

### Other variables

Other variables that might influence the relationship between severe maternal morbidity and psychological outcomes were defined as potential confounders, mediators and effect modifiers.

#### Potential confounders

Confounders are variables which are associated with both the risk factor and causally related to the outcome [36]. They may cause distortion in the effect of exposure of interest because “the effect of extraneous factors is mistaken for or mixed with the actual exposure effect (which may be null)” [37], p.120.

Following a systematic review [10], we included socio-demographic characteristics (maternal age, parity, ethnicity, educational qualification and an Index of Multiple Deprivation (IMD) [38]) and pre-existing health conditions (BMI as a measure of the level of overweight or obesity, and self-reported mental health history identified prior to giving birth) as potential confounders. Although a number of maternal health conditions prior to pregnancy may impact on a woman’s experiences of severe maternal morbidity [39-42], BMI and self-reported mental health history were selected because they are not only associated with severe maternal morbidity, but may be potential risk factors for psychiatric disorder [43]. Woman’s self-reported mental health history was treated as a binary variable with possible responses ‘Yes’ or ‘No’. Women were classified as part of the ‘Yes’ group if they had at least one of the following mental health problems at the time of their maternity booking for the index pregnancy: 1) A history of schizophrenia, bipolar affective disorder, depression or any other psychotic illness; 2) A history of postpartum psychotic illness (for multiparous women); 3) A history of inpatient or outpatient treatment by a psychiatrist or mental health team; 4) Self-reported feelings of feeling ‘down’ , depressed or hopeless and/or with ‘little interest or pleasure in doing things’ during pregnancy (in the past month); 5) A family history of severe mental illness in the postnatal period; 6) A family history of bipolar affective disorder (manic depression). Woman’s self-reported mental health history was considered to be ‘No’ if the woman did not report any of the above conditions.

#### Potential mediators

A mediator is a variable “which represents the generative mechanism through which the focal independent variable [i.e., the exposure of interest] is able to influence the dependent variable of interest [i.e., the outcome]” [44], p.1173. Rothman and Greenland [37] suggested that any factor that could be a step in the causal chain between exposure and disease should be treated not as a confounder but as a mediator (an intermediate variable). Several statisticians [45,46] have argued that variables which may act as potential mediators (or variables on the causal pathway to the outcome) should not be adjusted for as this may also adjust away the effect of the exposure of interest (“over-adjustment” [45], p.76).

Based on systematic reviews [10,47], we included a measure of women’s perceived control during labour and birth using the Labour Agentry Scale (LAS), in which higher scores indicate greater control [48]. The construct validity of the LAS has also been supported by evidence from a series of studies [48-50]. In the current study, the LAS showed good internal consistency reliability with a Cronbach’s alpha of 0.82. We also included neonatal outcomes (gestational age at birth, infant birth weight, infant Apgar score at 1 and 5 minutes, and neonatal intensive care unit admission), medical intervention during labour and birth (mode of birth and manual removal of placenta), and place of birth as potential mediators.

#### Potential effect modifiers

Effect modification (which is often termed ‘interaction’) occurs “when the impact of a risk factor on the outcome is changed by the value of a third variable” [45], p.11. The most central difference between effect modification and confounding is that “whereas confounding is a bias that the investigator hopes to prevent or remove from the effect estimate”, effect modification is a real effect and “a property of the effect under study” which the investigator wants to report in the findings [37], p.254.

We included variables to measure social support and perceived stressful events during the 6–8 week postnatal period as potential effect modifiers [45]. Postnatal social support was measured by a woman’s living arrangements and the Social Support Scale (SSS), a self-report scale with evidence of sufficient construct validity based on a sample of women from the Avon Longitudinal Study of Pregnancy and Childhood (ALSPAC) [51-54]. To understand women’s perceived stress during the postpartum period as a possible consequence of events other than giving birth, we also included the question, “Aside from your birth, have you experienced any changes in your life within the last six weeks, which have caused you anxiety or depression?” If their answer was positive, women were asked to report the event they had experienced. As these variables were measured during the postnatal period, they were not considered as potential confounders. For this reason, they were not included when adjusting for women’s baseline characteristics.

### Setting and participants

The site was one of the largest inner city maternity units in England serving a diverse population of women. Women who gave birth under the care of the unit between 7th June and 21st December 2010 were invited to participate. Women who booked to receive their maternity care at the unit could receive obstetric led care (care provided in the main unit, with obstetricians taking primary responsibility for women at high risk of obstetric complications, and midwives taking primary responsibility for women at low risk); care in a midwifery led birth-centre (located in the main unit, with midwives taking primary responsibility for care); or planned home birth (care for by community-based midwives employed by the unit). The inclusion criterion was all women who gave birth after 24 weeks gestation regardless of place of birth, which included women who planned to give birth at the unit but had an unplanned home birth. Exclusion criteria were women unable to read or understand English, women under 16 years old and those who experienced a stillbirth or neonatal death.^a^ Full ethics and R&D approval were obtained from the NHS Research Ethics Committee (REC 10/H0772/15) and the study site.

The sample size was based on PTSD symptoms measured by a total score of 20 or more on *both* the intrusion *and* avoidance subscales, which was one of the secondary outcomes. We considered all of the four dimensions of PTSD symptoms (≥20 on the IES intrusion subscale, ≥20 on the avoidance subscale, ≥20 on *either* the intrusion *or* avoidance subscales, and ≥20 on *both* the intrusion *and* avoidance subscales) to be important. Thus, it was essential to have a sufficient sample size to give 80% power for the detection of a significant (at the 5% level) difference in PTSD symptoms among women who did or did not experience severe morbidity for all of the dimensions of PTSD symptoms. PTSD symptoms that required a total score of 20 or more on *both* subscales needed the largest sample size. Therefore, we calculated the sample size based on this outcome. Czarnocka and Slade [8] found that approximately 2% of women from two hospitals in Sheffield, England, had clinically significant levels of both intrusion and avoidance as measured by the IES (≥20 on both subscales) at six weeks postpartum. A Dutch study [3] found that 28% of women had PTSD symptoms (using a self-reported measurement, the PTSD Symptom Scale) within 2 years following births complicated by severe pre-eclampsia, which is likely to account for the higher percentage. The estimate of the incidence of severe maternal morbidity was based on findings of Waterstone et al. [26], who found that 1.2% of women in their sample from the South East Thames region of England experienced severe maternal morbidity (defined as eclampsia, severe pre­eclampsia, HELLP syndrome, severe haemorrhage, severe sepsis, and uterine rupture). Based on these findings, a sample size of 1,585 was required, and allowing for a 50% loss to follow-up after excluding ineligible women, a total of 3,170 women who met the inclusion criterion were needed to participate.

### Data collection

Midwives provided a study information package to all women who met the study inclusion criterion before they were discharged home from the postnatal ward. Women who gave birth at home were provided with the information package by their community midwives. The package included an invitation letter with a study opt-out sheet and a research information leaflet. Women who did not wish to take part were asked to return the opt-out slip before receiving a questionnaire. All women who did not return an opt-out slip were informed that they could withdraw at anytime during the study. Following cognitive testing [55] with a small number of postnatal women (n = 4), information on postnatal outcomes, including PTSD symptoms, depression and other variables—such as women’s perceived control during labour and birth and perceived social support and stressful events during the postnatal period—was obtained from a follow-up questionnaire posted to women between 6 and 8 weeks after they gave birth. Women were asked to return the questionnaire with a signed consent form in order to participate in the study. A reminder was sent two weeks after the first mailing.

Information on baseline characteristics, pregnancy, birth and neonatal outcomes (including incidence of severe maternal morbidity) of all women who met inclusion criteria was extracted from electronic inpatient maternity records by the IT manager and consultant midwives in the study site (datasets did not include any personally identifiable data except for a study ID). Data from the maternity records were then merged with data from the postnatal questionnaire. Following this, all identifiable data on women who did not return the postnatal questionnaire or did not provide consent for their maternity records to be accessed were removed. This dataset was used to compare baseline characteristics of respondents and non-respondents. A separate dataset was then created that only included data from women who gave consent for their maternity records to be accessed.

### Statistical analysis

Data analysis was undertaken using SPSS v.19. Descriptive statistics were obtained on postnatal PTSD symptoms and symptoms of depression at 6–8 weeks postpartum. Postnatal outcomes were initially compared between women with and without severe maternal morbidity using Pearson’s chi-square tests, Fisher’s exact tests and T-tests as appropriate. Multivariable logistic regression models were developed to examine the relationship between severe maternal morbidity and primary outcomes adjusting for women’s baseline characteristics. Following recommendations of Baron and Kenny [44], the mediation analysis first tested the relationship between severe maternal morbidity (exposure) and a potential mediator (ie. perceived control during labour and birth measured by the total score of the LAS, neonatal outcomes, mode of birth and place of birth). Next, the bivariate relationship between the potential mediator and PTSD symptoms was examined. If the potential mediator showed statistical significance with both severe maternal morbidity and PTSD symptoms, multivariable logistic regression models were developed to see if the effect size of severe maternal morbidity on PTSD symptoms disappeared (fully mediated) or were reduced (partially mediated) by adding the potential mediator. Logistic regression models were also used to examine a possible effect modification of postnatal social support and other perceived stressful events respectively, on the relationship between severe maternal morbidity and PTSD symptoms, using interaction terms. If the results did not indicate the presence of effect modification (in other words, interaction terms were not significantly associated with PTSD symptoms), they were treated as potential risk factors and simply adjusted for in the multivariable logistic regressions model without using the interaction term. Pairwise deletion was performed for missing data.

## Results

**Figure 1:**
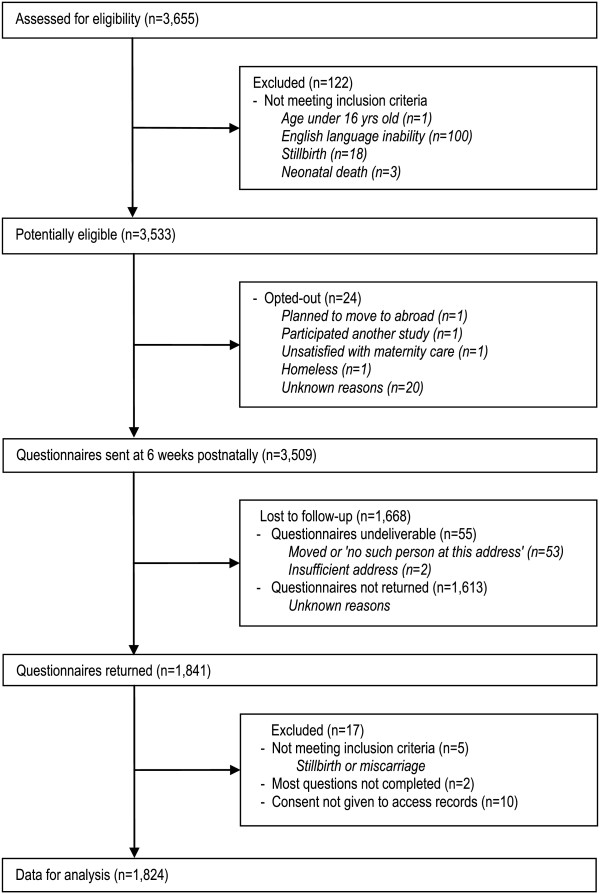
Flow of participants through the study.

Figure 1 shows the flow of participants through the study. Of the potentially eligible women (n = 3,533), 24 women opted out. In total, questionnaires were sent to 3,509 women at 6 – 8 weeks after giving birth. Fifty-five women could not be contacted by mail. A total of 1,841 questionnaires were returned, although 17 had to be excluded because they were completed by women who had suffered a stillbirth or miscarriage (n = 5), most questions were not completed (n = 2) or consent to access clinical records was not provided (n = 10). The final response rate was 53% (n = 1824), excluding the 55 women from the denominator (therefore, 52% of all eligible women). Time of questionnaire completion ranged from 6 to 16 weeks, the majority (74.2%) completing the questionnaire at 6–8 weeks postnatally; 93% completed within 10 weeks.

### Sample characteristics and severe maternal morbidity

Respondents were older, more likely to be primiparous, of white ethnicity and living in less-deprived areas compared to non-responders. There were significantly more instrumental and fewer spontaneous vaginal births (SVD) in respondents than non-respondents although rates of caesarean birth (either elective or emergency) were similar. There were no differences between respondents and non-respondents in severe maternal morbidity exposure where data were available (Additional file 1).

Of the study respondents, 147 (8.1%) experienced severe maternal morbidity based on our definition of this (Table 1).

**Table 1:** Severe maternal morbidity (Respondents = 1,824)

**Severe maternal morbidity**	**N**
**Major obstetric haemorrhage**[25]	73
Estimated blood loss ≥1500 ml (either vaginal or caesarean section related), or transfused 4 or more units of blood during labour, birth or immediately after birth	
**Eclampsia**[56]	4
A convulsive condition associated with pre-eclampsia	
**Severe pre-eclampsia**[57]	7
Pre-eclampsia with an existence of blood pressure of 160/110 mmHg	
**HELLP syndrome**[25]	1
Haemolysis (abnormal peripheral blood smear or raised total bilirubin concentration (>20.5 μmol/l)), raised liver enzyme activity (raised aspartate aminotransferase (>70 U/l)) or raised γglutamyltransferase (>70 U/l), and low platelets (<100 × 10^9^/l))	
**Intensive care unit (ICU)/High dependency unit (HDU) admission**	
ICU/HDU admission after giving birth. Admission for one of the above conditions or for any other reason.	103
**Total (All severe maternal morbidity cases)**	**147**

Numbers do not add up to n = 147 because some women had more than one condition.

### Prevalence of postnatal PTSD symptoms and other psychological outcomes

Descriptive statistics of each postnatal outcome are presented in Table 2.

**Table 2:** Postnatal outcomes

	**Frequency**	**Percentage**	**95%CI**
**Prevalence of PTSD symptoms**			
**• *Intrusion subscale***			
<20 on IES Intrusion	1669	93.6%	--
≥20 on IES Intrusion	114	6.4%	5.3-7.5
(missing)	(41)		
**• *Avoidance subscale***			
<20 on IES avoidance	1631	91.6%	--
≥20 on IES avoidance	150	8.4%	7.1-9.7
(missing)	(43)		
**• *Either intrusion OR avoidance subscale***			
<20 on *both* IES subscales	1562	88.5%	--
≥20 on *either* IES Intrusion *or* ≥20 IES avoidance	203	11.5%	10.0-13.0
(missing)	(59)		
***Both intrusion AND avoidance subscales***			
<20 on at least one IES subscale	1704	96.5%	--
≥20 on *both* IES intrusion *and* ≥20 IES avoidance	61	3.5%	2.6-4.3
(missing)	(59)		
**PTSD symptoms (continuous)**			
IES intrusion scores	mean = 5.79	sd = 7.33	5.5-6.2
IES avoidance scores	mean = 5.36	sd = 7.79	5.0-5.7
IES total scores	mean = 11.13	sd = 13.98	10.5-11.8
**Prevalence of probable depression**			
EPDS < 13	1,535	86.0%	--
EPDS ≥ 13	250	14.0%	12.4-15.6
(missing)	(39)		
**Depressive symptoms (continuous)**			
EPDS total scores	mean = 6.76	sd = 5.10	6.53-7.00
**Total**	**1,824**		

*IES* Impact of Event Scale, *EPDS* Edinburgh Postnatal and Depression Scale.

### PTSD symptoms and other psychological outcomes in women with and without severe maternal morbidity

Bivariate analysis showed that the proportion of subjects having PTSD symptoms was statistically significantly higher for women with severe maternal morbidity than women without severe maternal morbidity. The results were consistent for four indicators of PTSD symptoms: (1) intrusion (primary outcome), (2) avoidance (primary outcome), (3) *either* intrusion *or* avoidance (secondary outcome), and (4) *both* intrusion *and* avoidance (secondary outcome) (see Table 3). The difference in the mean score of the IES was also statistically significant, indicating that women with severe maternal morbidity had more frequent symptoms of intrusion and avoidance at 6 to 8 weeks postpartum. However, no statistically significant differences were observed in either prevalence or severity of depressive symptoms.

**Table 3:** PTSD symptoms and other psychological outcomes in women with and without severe maternal morbidity

	**No severe maternal morbidity**		**Severe maternal morbidity**		
	**N, mean**	**%, sd**	**N, mean**	**%, sd**	**P**
**Prevalence of PTSD symptoms**					
• *Intrusion subscale*					
<20 on IES Intrusion	1,541	94.1%	128	88.3%	<0.01
≥20 on IES Intrusion	97	5.9%	17	11.7%	
(missing)	(39)	--	(2)	--	
• *Avoidance subscale*					
<20 on IES avoidance	1,518	92.7%	113	79.0%	<0.001
≥20 on IES avoidance	120	7.3%	30	21.0%	
(missing)	(39)	--	(4)	--	
• *Either intrusion OR avoidance subscale*					
<20 on *both* IES subscales	1,454	89.6%	108	75.5%	<0.001
≥20 on *either* IES Intrusion *or* ≥20 IES avoidance	168	10.4%	35	24.5%	
(missing)	(55)	--	(4)	--	
• *Both intrusion AND avoidance subscales*					
<20 on at least one IES subscale	1,573	97.0%	131	91.6%	0.003
≥20 on *both* IES intrusion *and* ≥20 IES avoidance	49	3.0%	12	8.4%	
(missing)	(55)	--	(4)	--	
**PTSD symptoms (continuous)**					
IES intrusion scores	mean = 5.50	sd = 7.16	mean = 9.05	sd = 8.41	<0.001
IES avoidance scores	mean = 5.07	sd = 7.57	mean = 8.71	sd = 9.35	<0.001
IES total scores	mean = 10.54	sd = 13.61	mean = 17.79	sd = 16.33	<0.001
**Prevalence of probable depression**					
<13 on EPDS	1,412	86.0%	123	85.4%	0.90
≥13 on EPDS	229	14.0%	21	14.6%	
(missing)	(36)	--	(3)	--	
**Depressive symptoms (continuous)**					
EPDS total scores	mean = 6.72	sd = 5.11	mean = 7.30	sd = 4.90	0.19
**Total**	**1,677**		**147**		

### Relationship between severe maternal morbidity and PTSD symptoms

Multivariable logistic regression was developed for the primary outcomes (ie. PTSD symptoms of intrusion and avoidance), following bivariate analysis which examined the relationship of women’s baseline characteristics with severe maternal morbidity exposure (Additional file 2) and the outcome (Additional file 3). Results showed that women with severe maternal morbidity had significantly higher odds of having intrusion and avoidance when compared to women without severe maternal morbidity, even after adjusting for women’s baseline characteristics (data not presented).

A series of multivariable logistic regression models were then developed to assess whether the relationship between severe maternal morbidity and PTSD symptoms was mediated by the women’s perceived control during labour and birth (measured by the total score of the LAS), infant Apgar score at 5 minutes, mode of birth and place of birth. We selected these four variables following bivariate analysis. The first model (Model 1 in Table 4) shows unadjusted odds ratios for the relationship between severe maternal morbidity and each of the two indicators of PTSD symptoms, intrusion and avoidance. The second model (Model 2 in Table 4) adjusted for clinically important baseline characteristics; age, parity, ethnicity and BMI (potential risk factors of poorer health outcomes), although none met the criteria to be confounders from a statistical point of view. Model 3A in Table 4 showed that the relationship between severe maternal morbidity and PTSD symptoms remained statistically significant (p = 0.023 and <0.001 for intrusion ≥ 20 and avoidance ≥ 20, respectively) once the effect of women’s perceived control during labour and birth on PTSD symptoms was removed, although the effect was reduced (from OR = 2.24 to 2.04 for ≥20 on IES intrusion subscale; from OR = 3.38 to 3.15 for ≥20 on avoidance subscale). A similar analysis showed that although better neonatal outcomes (Model 3B) and/or no emergency caesarean birth (Model 3C) slightly reduced the effect of SMM on avoidance symptoms, any mediation effects were partial. There was no evidence that the relationship between SMM and PTSD symptoms was mediated by place of birth (Model 3D). Results consistently showed a direct, statistically significant association between severe maternal morbidity and PTSD symptoms at 6 – 8 weeks postpartum.

**Table 4:** Multivariable logistic regression models: association between severe maternal morbidity and PTSD symptoms (mediation analyses)

		**≥20 on IES Intrusion subscale**			**≥20 on IES Avoidance subscale**		
		**ORs**	**(95%CI)**	**P**	**ORs**	**(95%CI)**	**P**
**Model 1**	**SMM (unadjusted)**						
	SMM vs. Non-SMM	2.24	(1.25-4.00)	0.007	3.23	(2.01-5.17)	<0.001
							
**Model 2**	**SMM (adjusted for potential confounders)**^†^						
	SMM vs. Non-SMM	2.23	(1.23-4.05)	0.008	3.37	(2.08-5.46)	<0.001
							
**Model 3A**	**SMM**^†^						
	SMM vs. Non-SMM	2.04	(1.10-3.75)	0.023	3.14	(1.90-5.16)	<0.001
	Women’s perceived control - LAS^†^ (unit = 1 score)	0.94	(0.93-0.96)	<0.001	0.95	(0.93-0.96)	<0.001
							
**Model 3B**	**SMM**^†^						
	SMM vs. Non-SMM	2.21	(1.22-4.01)	0.009	3.28	(2.02-5.33)	<0.001
	Apgar score at 5 min.^†^ (unit = 1 score)	0.90	(0.72-1.12)	0.34	0.76	(0.64-0.90)	0.002
							
**Model 3C**	**SMM**^†^						
	SMM vs. Non-SMM	2.14	(1.15-3.98)	0.017	2.55	(1.53-4.24)	<0.001
	**Mode of birth**^†^			*Overall 0.69*			*Overall <0.001*
	Assisted vaginal vs. SVD	0.76	(0.40-1.46)	0.42	0.94	(0.52-1.70)	0.83
	Elective CS vs. SVD	0.92	(0.43-1.94)	0.82	1.61	(0.88-2.94)	0.12
	Emergency CS vs. SVD	1.17	(0.68-1.99)	0.57	2.43	(1.56-3.80)	<0.001
							
**Model 3D**	**SMM**^†^						
	SMM vs. Non-SMM	2.16	(1.18-3.94)	0.012	3.25	(1.99-5.29)	<0.001
	**Place of birth**^†^			*Overall 0.12*			*Overall 0.31*
	AMU vs. OU	1.05	(0.59-1.85)	0.88	0.93	(0.56-1.53)	0.77
	Planned home vs. OU	--	--	--	0.32	(0.04-2.33)	0.26
	BBA vs. OU	3.26	(1.28-8.33)	0.013	1.97	(0.78-4.98)	0.15

*SMM* Severe maternal morbidity, *LAS* Labour Agentry Scale.

^†^Adjusted for age, parity, ethnic groups, BMI. The results for these variables have been omitted from Models 2, 3A, 3B, 3C and 3D for simplicity of presentations.

-- There were too few cases of intrusion to calculate the OR.

Finally, we developed a multivariable logistic regression model following bivariate analyses to assess the association between severe maternal morbidity and PTSD symptoms, taking into account levels of social support and perceived stressful events during the 6–8 week postnatal period. Since there was no evidence that either of these were effect modifiers (eg. interaction terms were not statistically significant), these factors were simply adjusted for. A statistically significant difference between severe maternal morbidity and PTSD symptoms remained (Table 5). Living arrangements were not included in the model as these were not associated with severe maternal morbidity or PTSD symptoms.

**Table 5:** **Multivariable logistic regression model: association between severe maternal morbidity and PTSD symptoms adjusted for potential confounders**^†^

	**≥20 on IES Intrusion subscale**			**≥20 on IES Avoidance subscale**		
	**ORs**	**(95%CI)**	**P**	**ORs**	**(95%CI)**	**P**
**SMM**						
SMM vs. Non-SMM	2.21	(1.24-3.96)	0.007	3.58	(2.20-5.84)	<0.001
**Perceived social support - SSS**						
(unit = 1 score)	0.97	(0.93-1.00)	0.06	0.91	(0.88-0.94)	<0.001
**Perceived stressful event**						
Yes vs. No	1.61	(0.94-2.77)	0.08	1.24	(0.75-2.03)	0.40

*SMM* Severe maternal morbidity, *SSS* Social Support Scale.

^†^Adjusted for age, parity, ethnic groups, BMI. The results for these variables have been omitted from the model for simplicity of presentation.

## Discussion

This is one of the largest studies to date to have examined PTSD symptoms among women who have recently given birth and association with severe maternal morbidity. The prevalence of PTSD symptoms at 6 – 8 weeks postpartum was within the range estimated from previous studies. Although relatively large numbers of women experienced PTSD symptoms irrespective of severe maternal morbidity, this study found evidence of a higher risk of PTSD symptoms among women who experienced severe maternal morbidity compared with women who did not, in one inner city area of England. The current study also found that a higher level of women’s perceived control during labour and birth potentially reduced the effect of severe maternal morbidity on PTSD symptoms. This finding supports the recent synthesis of qualitative studies of women’s experiences and perceptions of severe maternal morbidity [47] that showed clinical care and the organisation of care (e.g., communication with healthcare professionals) could either mitigate or worsen the negative effects of severe maternal morbidity. These negative effects include women’s feelings of loss of control, which can in turn, affect the development of PTSD symptoms. In contrast to PTSD symptoms, there was no evidence of an association between severe maternal morbidity and probable depression as measured using the EPDS, consistent with a previous matched cohort study of severe maternal morbidity conducted with women in the same region [22] and two smaller studies from the Netherlands [3,58]. This may be because, unlike PTSD, in which there is almost always a precipitating event [59], depression often occurs without a specific trigger. This is reflected in the DSM-IV, in which PTSD is described as one of only a few mental disorders for which there is a known cause. In contrast, “a diagnosis of depression opens the issue of causation to many factors other than the stated cause of action” [60], p.297. Therefore any relationship between severe maternal morbidity and depression could be obscured given the many possible causes of depression post-birth.

### Strengths and limitations

#### Strengths

The study design overcame methodological limitations of previous studies such as small sample size [3,58,61,62] and unclear definition of severe maternal morbidity [2,30,63,64]. In previous studies with comparatively small samples, the association between SMM and PTSD was often investigated by exploring differences in the mean score of the self-reported measurement of the PTSD symptoms between the risk and non-risk groups. Since the current study had a relatively large sample size, it was possible to compare the proportion of women with a clinically significant level of PTSD symptoms among those with and without severe maternal morbidity. Moreover, in this prospective study, severe maternal morbidity was identified from women’s maternity records to minimise recall bias. An additional strength was that the variables potentially on the causal pathway between SMM and PTSD symptoms (ie. women’s perceived control during labour and birth, neonatal outcomes, mode of birth, place of birth) were treated as potential mediators. This was important because in previous studies, these variables were simply adjusted for and by doing so the potential effect of severe maternal morbidity might have been eliminated [45].

#### Limitations

While avoiding over-adjustment [45] was important, mediation analysis was based on the assumption that severe maternal morbidity might affect PTSD symptoms through potential mediators. However the direction of the relationship between severe maternal morbidity and the mode and place of birth could go either way (eg. mode and/or place of birth might be pre-selected because of severe maternal morbidity or severe maternal morbidity might occur because of mode and/or place of birth). If the latter, then mode and place of birth could be confounders rather than mediators. Because confounders and mediators are statistically very similar, it was not possible to determine the true mechanism of the relationship between severe maternal morbidity and PTSD symptoms. The statistical significance remained the same, however, regardless of whether mode and place-of-birth variables were included in the model. Therefore, the significant association between severe maternal morbidity and PTSD symptoms was unlikely to be altered by inclusion of these variables in the model.

Another limitation is that women’s perceived control during labour and birth and perceived social support were measured postnatally and it is again difficult to apportion cause and effect. There is a possibility that the association between these variables and PTSD symptoms could be attributed to recall bias in which women with PTSD symptoms were more likely to remember feelings of fear, helplessness, and/or being uncared for during their labour and birth. Similarly, women with PTSD might have felt a lack of support because they needed more support than those with no symptoms. If this was the case, it would be incorrect to include these variables in logistic regression models. The results of multivariable logistic regression analysis with and without these variables, however, did not change the significant association between severe maternal morbidity and PTSD symptoms indicating that study results were unlikely to be affected.

Although higher rates of women’s perceived control during labour and birth appeared to reduce the effect of severe maternal morbidity on PTSD symptoms, statistical data itself did not permit an understanding of how much, as it was measured as a continuous score using the LAS. Postnatal outcomes were also collected using a self-administered questionnaire. The measures used were carefully selected, with published accounts of their validity and reliability taken into consideration. Nevertheless, the identification of diagnostic PTSD and depression was not possible in this study. Moreover, PTSD symptoms might vary by the time of questionnaire completion. However due to the small proportion of women who completed the questionnaire very late (> 10 weeks), it was difficult to examine this.

Although we included the women’s self-reported mental health histories as collected from maternity booking records, we were unable to measure other maternal characteristics such as previous traumatic events before birth (childhood trauma including previous abuse) and personality type. Individuals with PTSD symptoms might suffer intrusive and distressing memories of past experiences triggered by the current stress event, during which time the individual confused the past stress with present circumstances [65]. Including such information could have been informative.

There is another limitation related to study generalisability. The study included women who had given birth under the care of one large inner city maternity unit and may only be generalisable to the population with similar demographic and obstetric characteristics. The numbers of women who were excluded was small, but it is possible that postnatal health issues were underestimated as a result of excluding potentially high-risk groups. The response rate was 52% of all eligible women, similar to the response rate in the same region (51%) in a recent national maternity survey [66,67]. Surveys demand literacy, engagement and organisation, and it was difficult to engage women from younger age groups, poorer areas or different ethnicities in this research, having significant differences between respondents and non-respondents. This is a common issue in research focusing on postnatal population in England as previous studies have shown [22,66].

Due to the lower response from more vulnerable groups and also because women with PTSD symptoms and/or depression might be less likely to respond, there is again a potential risk of the underestimation of psychological problems during the postnatal period as mentioned earlier. However, the results of the significant association between severe maternal morbidity and PTSD were less likely to be affected because the sample was relatively representative in terms of major obstetric haemorrhage (the majority cases of severe maternal morbidity) and none of the indicators for demographic characteristics were likely to be acting as confounders from a statistical point of view.

### Further analyses

It was notable from this study that of the women who did not experience severe maternal morbidity, 5.9% had a high score on intrusion, 7.3% on avoidance and 10.4% had either symptoms. This raises the questions regarding what factors contribute to PTSD symptoms, irrespective of severe maternal morbidity. To answer the question was beyond our original study aims, but will form the basis of a further secondary analysis of this data.

## Conclusions

Despite the concern about increases in the incidence of severe maternal morbidity little was known about the impact on women’s psychological health following birth. By conducting a prospective observational study we found clear relationship between women’s experiences of severe maternal morbidity and PTSD symptoms at 6–8 weeks. It is important to raise awareness about the relationship amongst women, clinicians and policy makers in order to prevent and manage severe maternal morbidity and its subsequent issues, and maximising use of finite resources.

In the current UK system of postnatal care, PTSD symptoms among women who have recently given birth may remain ‘hidden’ , for numerous reasons. PTSD is a recent concept, not currently routinely screened for by relevant health professionals. Timely and appropriate treatment of PTSD symptoms may not be offered to women due to the frequent misuse of the term ‘postnatal depression’ by health professionals as a label for any mental illness occurring postnatally [68]. Furthermore women may not report symptoms, due to concerns about social stigma or because they are unaware of the importance of seeking urgent professional support when they experience such symptoms. As recommended in the National Institute for Health and Clinical Excellence [69] guidelines on routine postnatal care, all women should be offered relevant information to recognise symptoms and signs of serious postnatal health problems that they may experience, including PTSD symptoms. Women should also be offered an opportunity to talk about their birth experiences and ask questions about the care they received during labour [69]. These are crucial issues particularly for women who experience severe maternal morbidity who may expect health professionals to help them to make sense of their experiences and the care they received to manage the condition [70].

More studies are required to establish what interventions would increase women’s perceived control when emergencies and severe complications occur. However, evidence from qualitative studies show that women feel more in control, even in an emergency situation, when informed about treatment options and involved in decision-making if possible [71,72]. It is therefore important to clearly communicate with women and their partners during an event; respecting their views and providing information and opportunity for them to ask and understand reasons for urgent medical treatment, which may make a difference to the subsequent impact of PTSD symptoms. More research is also needed to consider what care should be included during the shorter and longer-term postnatal period to minimise the impact of severe maternal morbidity as well as analyses of the cost-effectiveness of the proposed content and organisation of care.

### Ethical approval

Approval was obtained from the Camden & Islington Community Research Ethics Committee (REC 10/H0772/15) and study site. Written consents were also obtained from participants.

## Endnote

^a^The original intention was to include women who suffered a stillbirth or neonatal death because these women were thought to be more vulnerable to psychological problems including PTSD symptoms. During the first two months of recruitment, two women who had experienced a stillbirth unfortunately did not return a study opt-out letter and contacted the researcher expressing concern that they had received a postnatal questionnaire. These two women were excluded. The study team decided to exclude women who had stillbirth or neonatal death given concerns about the distress of being asked to participate. This amendment was approved by the ethics committee on 25 November 2010 (reference number: 10/H0722/15).

## Competing interests

The authors declare that they have no competing interests.

## Authors’ contributions

MF developed the protocol with the support of DB and JS. All authors contributed to the development of analysis plan. MF collected data with support from DB and JS as well as midwives and IT managers at the study site. MF and DK conducted the main analyses. All authors checked the results. All authors contributed to the manuscript. All authors read and approved the final manuscript.

## Pre-publication history

The pre-publication history for this paper can be accessed here:

http://www.biomedcentral.com/1471-2393/14/133/prepub

## Supplementary Material

Additional file 1: Table S1 Socio-demographic characteristics and pregnancy outcomes (respondents vs. non-respondents). Click here for file

Additional file 2: Table S2 Bivariate association between women’s baseline characteristics and severe maternal morbidity. Click here for file

Additional file 3: Table S3a Bivariate association between women’s baseline characteristics and PTSD symptoms (≥20 on intrusion subscale of the IES). **Table S3b** Bivariate association between women’s baseline characteristics and PTSD symptoms (≥20 on avoidance subscale of the IES). Click here for file
