# Supplementary material for: The relationship between severe maternal morbidity and psychological health symptoms at 6–8 weeks postpartum: a prospective cohort study in one English maternity unit
Source: BMC Pregnancy Childbirth. 2014 Apr 7;14:133. doi: 10.1186/1471-2393-14-133 (PMC4021064; doi:10.1186/1471-2393-14-133)
Supplement: Additional file 1: Table S1 — Socio-demographic characteristics and pregnancy outcomes (respondents vs. non-respondents). [file 1471-2393-14-133-S1.doc]

**Additional file 1**

Table S1 Socio-demographic characteristics and pregnancy outcomes (respondents vs. non-respondents)

|  | **All** | | | **Respondents** | | | **Non-respondents** | | |  |
| --- | --- | --- | --- | --- | --- | --- | --- | --- | --- | --- |
|  | N, mean | %, SD | N, mean | | %, SD | N, mean | | %, SD | **P** | |
| **Age at delivery (cont.)** | mean=31.3 | SD=5.65 | mean=32.3 | | sd=5.25 | mean=30.3 | | sd=5.88 | <0.001 | |
| **Age at delivery** |  |  |  | |  |  | |  |  | |
| ≤19 | 72 | 2.1%) | 21 | | 1.2% | 51 | | 3.0% | <0.001 | |
| 20-24 | 404 | 11.5% | 142 | | 7.8% | 262 | | 15.5% |  | |
| 25-29 | 736 | 21.0% | 328 | | 18.0% | 408 | | 24.2% |  | |
| 30-34 | 1,269 | 36.2% | 717 | | 39.3% | 552 | | 32.8% |  | |
| 35-39 | 810 | 23.1% | 491 | | 26.9% | 319 | | 18.9% |  | |
| 40+ | 218 | (6.2% | 125 | | 6.9% | 93 | | 5.5% |  | |
| (missing) | (0) | -- | (0) | | -- | (0) | | -- |  | |
| **Parity** |  |  |  | |  |  | |  |  | |
| Primiparous | 2,091 | 59.8% | 1,182 | | 64.8% | 909 | | 54.4% | <0.001 | |
| Multiparous | 1,404 | 40.2% | 642 | | 35.2% | 762 | | 45.6% |  | |
| (missing) | (14) |  | (0) | |  | (14) | |  |  | |
| **Ethnicity** |  |  |  | |  |  | |  |  | |
| White | 1,790 | 51.2% | 1,103 | | 60.5% | 687 | | 41.2% | <0.001 | |
| Black | 1,111 | 31.8% | 432 | | 23.7% | 679 | | 40.7% |  | |
| Asian | 306 | 8.8% | 158 | | 8.7% | 148 | | 8.9% |  | |
| Mixed/multiple | 86 | 2.5% | 45 | | 2.5% | 41 | | 2.5% |  | |
| Other | 200 | 5.7% | 86 | | 4.7% | 114 | | 6.8% |  | |
| (missing) | (16) |  | (0) | |  | (16) | |  |  | |
| **Highest education qualification** |  |  |  | |  |  | |  |  | |
| None | -- | -- | 86 | | 4.8% | -- | | -- |  | |
| GCSE | -- | -- | 207 | | 11.6% | -- | | -- |  | |
| A-level | -- | -- | 271 | | 15.1% | -- | | -- |  | |
| Degree/equivalent+ | -- | -- | 1,227 | | 68.5% | -- | | -- |  | |
| (Missing) |  |  | (33) | |  |  | |  |  | |
| **IMD** |  |  |  | |  |  | |  |  | |
| Least | 66 | 1.9% | 47 | | 2.6% | 19 | | 1.1% | <0.001 | |
| Fourth | 192 | 5.5% | 125 | | 6.9% | 67 | | 4.0% |  | |
| Third | 432 | 12.5% | 291 | | 16.1% | 141 | | 8.5% |  | |
| Second | 1,607 | 46.4% | 822 | | 45.6% | 785 | | 47.3% |  | |
| Most | 1,166 | 33.7% | 519 | | 28.8% | 647 | | 39.0% |  | |
| (missing) | (46) |  | (20) | |  | (26) | |  |  | |
| **BMI (kg/m^2^, cont.)** | -- | -- | mean=24.4 | | sd=4.92 | -- | | -- |  | |
| **BMI (kg/m^2^)** |  |  |  | |  |  | |  |  | |
| <18.5 | -- | -- | 47 | | 2.6% | -- | | -- |  | |
| 18.5-24.9 | -- | -- | 1,129 | | 63.5% | -- | | -- |  | |
| 25.0-29.9 | -- | -- | 401 | | 22.6% | -- | | -- |  | |
| 30.0-34.9 | -- | -- | 141 | | 7.9% | -- | | -- |  | |
| 35.0-39.9 | -- | -- | 37 | | 2.1% | -- | | -- |  | |
| ≥40.0 | -- | -- | 22 | | 1.2% | -- | | -- |  | |
| (missing) |  |  | (47) | |  |  | |  |  | |
| **Mental health history** |  |  |  | |  |  | |  |  | |
| No | -- | -- | 1,725 | | 96.0% | -- | | -- |  | |
| Yes | -- | -- | 72 | | 4.0% | -- | | -- |  | |
| (missing) |  |  | (27) | |  |  | |  |  | |
| **Total** | **3,509** |  | **1,824** | |  | **1,685** | |  |  | |

|  | **All** | | | **Respondents** | | | **Non-respondents** | | |  |
| --- | --- | --- | --- | --- | --- | --- | --- | --- | --- | --- |
|  | N, mean | %, SD | N, mean | | %, SD | N, mean | | %, SD | **P** | |
| **Mode of birth** |  |  |  | |  |  | |  |  | |
| SVD | 1,988 | 57.3% | 1,003 | | 55.0% | 985 | | 60.0% | <0.001 | |
| Breech/instrumental | 485 | 14.0% | 299 | | 16.4% | 186 | | 11.3% |  | |
| ElCS | 326 | 9.4% | 163 | | 8.9% | 163 | | 9.9% |  | |
| EmCS | 668 | 19.3% | 359 | | 19.7% | 309 | | 18.8% |  | |
| (missing) | (42) |  | (0) | |  | (42) | |  |  | |
| **Manual removal of placenta** |  |  |  | |  |  | |  |  | |
| No | -- | -- | 1,270 | | 69.6% | -- | | -- |  | |
| Manual removal | -- | -- | 32 | | 1.8% | -- | | -- |  | |
| N/A (c-section) | -- | -- | 522 | | 28.6% | -- | | -- |  | |
| (missing) |  |  | (5) | |  |  | |  |  | |
| **Place of birth** |  |  |  | |  |  | |  |  | |
| OU | -- | -- | 1,388 | | 76.1% | -- | | -- |  | |
| AMU | -- | -- | 348 | | 19.1% | -- | | -- |  | |
| HB | -- | -- | 51 | | 2.8% | -- | | -- |  | |
| BBA | -- | -- | 37 | | 2.0% | -- | | -- |  | |
| (missing) |  |  | (0) | |  |  | |  |  | |
| **Ges. age (weeks, cont)** |  |  | mean=39.2 | | sd=2.1 |  | |  |  | |
| **Ges. age (weeks)** |  |  |  | |  |  | |  |  | |
| <37 | 289 | 8.3% | 145 | | 7.9% | 144 | | 8.6% | 0.32 | |
| 37≤, <42 | 2,960 | 84.6% | 1,558 | | 85.4% | 1,402 | | 83.7% |  | |
| 42≤ | 251 | 7.2% | 121 | | 6.6% | 130 | | 7.7% |  | |
| (missing) | (9) |  | (0) | |  | (9) | |  |  | |
| **Birth weight (g. cont.)** |  |  | mean=3,363 | | sd=576.6 |  | |  |  | |
| **Birth weight (g)** |  |  |  | |  |  | |  |  | |
| <2500 |  |  | 116 | | 6.4% |  | |  |  | |
| 2500≤, <4500 |  |  | 1671 | | 91.9% |  | |  |  | |
| 4500≤ |  |  | 31 | | 1.7% |  | |  |  | |
| (missing) |  |  | (6) | |  |  | |  |  | |
| **Apgar at 1 min. (disc.)** |  |  | mean=8.6 | | sd=1.3 |  | |  |  | |
| **Apgar at 1 min.** |  |  |  | |  |  | |  |  | |
| 0-3 |  |  | 22 | | 1.2% |  | |  |  | |
| 4-6 |  |  | 113 | | 6.2% |  | |  |  | |
| 7-10 |  |  | 1,681 | | 92.6% |  | |  |  | |
| (missing) |  |  | (8) | | -- |  | |  |  | |
| **Apgar at 5 min. (disc)** |  |  | mean=9.7 | | sd=0.8 |  | |  |  | |
| **Apgar at 5 min.** |  |  |  | |  |  | |  |  | |
| 0-6 | 37 | 1.1% | 17 | | 0.9% | 20 | | 1.2% | 0.44 | |
| 7-10 | 3,446 | 98.9% | 1,800 | | 99.1% | 1,646 | | 98.8% |  | |
| (missing) | (26) |  | (7) | |  | (19) | |  |  | |
| **NICU admission** |  |  |  | |  |  | |  |  | |
| No |  |  | 1,735 | | 95.1% |  | |  |  | |
| NICU |  |  | 88 | | 4.8% |  | |  |  | |
| (missing) |  |  | (1) | | -- |  | |  |  | |
| **Total** | **3,509** |  | **1,824** | |  | **1,685** | |  |  | |

Ges. age=gestational age at birth, OU=Obstetric unit, AMU=Alongside midwifery unit, HB=Planned home birth, BBA=Birth before arrival, cont=continuous, disc=discrete, --=data unavailable

|  | **All** | | **Respondents** | | **Non-respondents** | |  |
| --- | --- | --- | --- | --- | --- | --- | --- |
|  | N, mean | %, SD | N, mean | %, SD | N, mean | %, SD | **P** |
| **Major obstetric haemorrhage (EBL)** |  |  |  |  |  |  |  |
| <1,500ml | 3,351 | 96.3% | 1,746 | 96.1% | 1,605 | 96.5% | 0.56 |
| ≥1,500ml | 128 | 3.7% | 70 | 3.9% | 58 | 3.5% |  |
| (missing) | (30) |  | (8)^†^ |  | (22) |  |  |
| **Major obstetric haemorrhage**  **(EBL & transfusion)** |  |  |  |  |  |  |  |
| <1,500ml &  transfusion ≤3 units | -- | -- | 1,743 | 96.0% | -- | -- | -- |
| ≥1,500ml or  transfusion 4+ units | -- | -- | 73 | 4.0% | -- | -- |  |
| (missing) |  |  | (8)^†^ | -- |  |  |  |
| **Eclampsia** |  |  |  |  |  |  |  |
| No | -- | -- | 1,820 | 99.8% | 1,685 | -- | -- |
| Yes | -- | -- | 4 | 0.2% | 0 | -- |  |
| (missing) |  |  | (0) |  | (0) |  |  |
| **HELLP syndrome** |  |  |  |  |  |  |  |
| No | 3,508 | -- | 1,823 | 99.9% | 1,685 | -- | -- |
| Yes | 1 | -- | 1 | <0.1% | 0 | -- |  |
| (missing) | (0) |  | (0) |  | (0) |  |  |
| **Admitted to the HDU** |  |  |  |  |  |  |  |
| No | -- | -- | 1,721 | 94.4% | -- | -- | -- |
| Yes | -- | -- | 103 | 5.6% | -- | -- |  |
| (missing) |  |  | (0) |  |  |  |  |
| **All severe maternal morbidity cases** |  |  |  |  |  |  |  |
| No | -- | -- | 1,677 | 91.9% | -- | -- | -- |
| Yes | -- | -- | 147 | 8.1% | -- | -- |  |
| (missing) |  |  | (0)^†^ |  |  |  |  |
| **Total** | **3,509** |  | **1,824** |  | **1,685** |  |  |

† Numbers were missing for the estimated blood loss, but none of these missing cases had blood transfusion. These missing cases were therefore put in the category of non- severe maternal morbidity group when the variable of ‘all severe maternal morbidity cases’ was created.
