# Supplementary material for: The relationship between severe maternal morbidity and psychological health symptoms at 6–8 weeks postpartum: a prospective cohort study in one English maternity unit
Source: BMC Pregnancy Childbirth. 2014 Apr 7;14:133. doi: 10.1186/1471-2393-14-133 (PMC4021064; doi:10.1186/1471-2393-14-133)
Supplement: Additional file 2: Table S2 — Bivariate association between women’s baseline characteristics and severe maternal morbidity. [file 1471-2393-14-133-S2.doc]

**Additional file 2**

Table S2 Bivariate association between women’s baseline characteristics and severe maternal morbidity

|  | **Frequency** | **ORs** | **95%CI** | **P** |
| --- | --- | --- | --- | --- |
| **Age at delivery** |  |  |  |  |
| Continuous, unit=year | 1824 | 1.06 | 1.03 to 1.10 | **0.001** |
| (missing) | (0) | -- | -- | -- |
| **Age-group** |  |  |  | ***Overall: 0.01*** |
| Under 20 | 21 | 2.03 | 0.39-10.50 | 0.40 |
| 20 - 24 | 142 | 1 | -- | -- |
| 25 - 29 | 328 | 0.92 | 0.37-2.32 | 0.87 |
| 30 - 34 | 717 | 1.67 | 0.74-3.73 | 0.22 |
| 35 - 39 | 491 | 2.09 | 0.92-4.73 | 0.08 |
| 40 + | 125 | 3.24 | 1.31-8.05 | 0.01 |
| (missing) | (0) | -- | -- | -- |
| **Parity** |  |  |  |  |
| Primiparity | 1184 | 1 |  | -- |
| Multiparity | 640 | 0.70 | 0.48-1.01 | **0.058** |
| (missing) | (0) | -- | -- | -- |
| **Ethnic groups** |  |  |  | ***Overall: 0.81*** |
| White | 1103 | 1 |  |  |
| Black | 432 | 0.90 | 0.59-1.36 | 0.61 |
| Asian | 158 | 0.97 | 0.53-1.79 | 0.93 |
| Mixed/Other | 131 | 0.71 | 0.34-1.49 | 0.71 |
| (missing) | (0) | -- | -- | -- |
| **Women’s education** |  |  |  | ***Overall: 0.46*** |
| None | 86 | 1 |  |  |
| GCSE | 207 | 0.62 | 0.26-1.49 | 0.29 |
| A-level | 271 | 0.57 | 0.25-1.34 | 0.20 |
| Degree and above | 1227 | 0.79 | 0.39-1.63 | 0.53 |
| (missing) | (33) | -- | -- | -- |
| **Deprivation quintiles (IMD)** |  |  |  | ***Overall: 0.13*** |
| Most | 520 | 1 |  | -- |
| Second | 822 | 1.49 | 0.99-2.26 | 0.06 |
| Third | 291 | 0.91 | 0.51-1.64 | 0.76 |
| Fourth | 125 | 0.82 | 0.36-1.90 | 0.65 |
| Least | 47 | 1.65 | 0.61-4.43 | 0.32 |
| (missing) | (19) | -- | -- | -- |
| **BMI** |  |  |  |  |
| Continuous, unit=1 kg/m^2^ | 1777 | 1.01 | 0.98-1.05 | **0.42** |
| (missing) | (47) | -- | -- | -- |
| **Mental health history** |  |  |  |  |
| No | 1725 | 1 |  |  |
| Yes | 72 | 0.49 | 0.15-1.56 | **0.22** |
| (missing) | (27) | -- | -- | -- |
| **Total** | **1824** |  |  |  |

Note: Reference groups were selected considering the sample size (the largest sample size) in the subgroups or the most (or the least) risk groups based on expectation.
