# Supplementary material for: The relationship between severe maternal morbidity and psychological health symptoms at 6–8 weeks postpartum: a prospective cohort study in one English maternity unit
Source: BMC Pregnancy Childbirth. 2014 Apr 7;14:133. doi: 10.1186/1471-2393-14-133 (PMC4021064; doi:10.1186/1471-2393-14-133)
Supplement: Additional file 3: Table S3a — Bivariate association between women’s baseline characteristics and PTSD symptoms (≥20 on intrusion subscale of the IES). Table S3b Bivariate association between women’s baseline characteristics and PTSD symptoms (≥20 on avoidance subscale of the IES). [file 1471-2393-14-133-S3.doc]

**Additional file 3**

Table S3_1 Bivariate association between women’s baseline characteristics and PTSD symptoms (≥20 on intrusion subscale of the IES)

|  | **Frequency** | **ORs** | **95%CI** | **P** |
| --- | --- | --- | --- | --- |
| **Age at delivery** |  |  |  |  |
| Continuous, unit=year | 1783 | 1.01 | 0.98 to 1.05 | 0.53 |
| (missing) | (41) |  |  |  |
| **Age-group** |  |  |  | ***Overall: 0.68*** |
| Under 20 | 19 | 1.70 | 0.34-8.53 | 0.52 |
| 20-24 | 139 | 1 |  |  |
| 25-29 | 318 | 0.92 | 0.41-2.08 | 0.84 |
| 30-34 | 701 | 0.92 | 0.44-1.94 | 0.83 |
| 35-39 | 483 | 0.96 | 0.44-2.07 | 0.91 |
| 40 + | 123 | 1.56 | 0.63-3.84 | 0.33 |
| (missing) | (41) | -- |  | -- |
| **Parity** |  |  |  |  |
| Primiparity | 1159 | 1 |  |  |
| Multiparity | 624 | 0.96 | 0.65-1.44 | 0.86 |
| (missing) | (41) | -- | -- | -- |
| **Ethnic groups** |  |  |  | ***Overall: 0.001*** |
| White | 1086 | 1 |  |  |
| Black | 417 | 2.07 | 1.35-3.17 | 0.001 |
| Asian | 151 | 0.81 | 0.34-1.91 | 0.63 |
| Mixed/Other | 129 | 2.57 | 1.40-4.70 | 0.002 |
| (missing) | (41) | -- | -- | -- |
| **Women’s education** |  |  |  | ***Overall: 0.42*** |
| None | 84 | 1 |  |  |
| GCSE | 201 | 0.48 | 0.19-1.21 | 0.12 |
| A-level | 262 | 0.54 | 0.23-1.28 | 0.16 |
| Degree and above | 1211 | 0.57 | 0.28-1.19 | 0.14 |
| (missing) | (66) | -- | -- | -- |
| **Deprivation quintiles (IMD)** |  |  |  | ***Overall: 0.08*** |
| Most | 505 | 1 |  |  |
| Second | 807 | 0.97 | 0.64-1.48 | 0.89 |
| Third | 285 | 0.54 | 0.28-1.05 | 0.07 |
| Fourth | 120 | 0.32 | 0.10-1.04 | 0.06 |
| Least | 47 | 0.27 | 0.04-1.99 | 0.20 |
| (missing) | (60) | -- | -- | -- |
| **BMI** |  |  |  |  |
| Continuous, unit=1 kg/m^2^ | 1738 | 1.06 | 1.02-1.09 | 0.001 |
| (missing) | (86) | -- | -- | -- |
| **BMI** |  |  |  | ***Overall: 0.001*** |
| <18.5 | 46 | 0.40 | 0.06-2.98 | 0.37 |
| 18.5-24.9 | 1111 | 1 |  |  |
| 25.0-29.9 | 386 | 1.20 | 0.74-1.97 | 0.46 |
| 30 + | 195 | 2.67 | 1.63-4.39 | <0.001 |
| (missing) | (86) | -- | -- | -- |
| **Mental health history** |  |  |  |  |
| No | 1688 | 1 |  |  |
| Yes | 71 | 1.63 | 0.73-3.65 | 0.23 |
| (missing) | (65) | -- | -- | -- |
| **Total** | **1824** |  |  |  |

Note: Reference groups were selected considering the sample size (the largest sample size) in the subgroups or the most (or the least) risk groups based on expectation.

Table S3_2 Bivariate association between women’s baseline characteristics and PTSD symptoms (≥20 on avoidance subscale of the IES)

|  | **Frequency** | **ORs** | **95%CI** | **P** |
| --- | --- | --- | --- | --- |
| **Age at delivery** |  |  |  |  |
| Continuous, unit=year | 1781 | 1.00 | 0.97 to 1.04 | 0.90 |
| (missing) | (43) |  |  |  |
| **Age-group** |  |  |  | ***Overall: 0.79*** |
| Under 20 | 20 | 0.54 | 0.07-4.39 | 0.64 |
| 20-24 | 135 | 1 |  |  |
| 25-29 | 319 | 1.14 | 0.57-2.30 | 0.71 |
| 30-34 | 703 | 0.84 | 0.43-2.62 | 0.59 |
| 35-39 | 481 | 0.93 | 0.47-1.83 | 0.83 |
| 40 + | 123 | 1.11 | 0.48-2.57 | 0.81 |
| (missing) | (43) | -- |  | -- |
| **Parity** |  |  |  |  |
| Primiparity | 1162 | 1 |  |  |
| Multiparity | 619 | 1.13 | 0.80-1.60 | 0.49 |
| (missing) | (43) | -- | -- | -- |
| **Ethnic groups** |  |  |  | ***Overall: 0.001*** |
| White | 1087 | 1 |  |  |
| Black | 413 | 2.08 | 1.43-3.02 | <0.001 |
| Asian | 154 | 1.08 | 0.56-2.09 | 0.81 |
| Mixed/Other | 127 | 1.75 | 0.95-3.20 | 0.07 |
| (missing) | (43) | -- | -- | -- |
| **Women’s education** |  |  |  | ***Overall: 0.002*** |
| None | 80 | 1 |  |  |
| GCSE | 201 | 0.73 | 0.35-1.51 | 0.40 |
| A-level | 259 | 0.27 | 0.12-0.62 | 0.002 |
| Degree and above | 1216 | 0.44 | 0.23-0.82 | 0.01 |
| (missing) | (68) | -- | -- | -- |
| **Deprivation quintiles (IMD)** |  |  |  | ***Overall: 0.24*** |
| Most | 501 | 1 |  |  |
| Second | 803 | 0.79 | 0.53-1.16 | 0.23 |
| Third | 288 | 0.52 | 0.29-0.93 | 0.027 |
| Fourth | 123 | 0.95 | 0.49-1.85 | 0.89 |
| Least | 47 | 1.05 | 0.40-2.78 | 0.92 |
| (missing) | (62) | -- | -- | -- |
| **BMI** |  |  |  |  |
| Continuous, unit=1 kg/m^2^ | 1737 | 1.04 | 1.01-1.07 | 0.007 |
| (missing) | (87) | -- | -- | -- |
| **BMI** |  |  |  | ***Overall: 0.02*** |
| <18.5 | 47 | 1.23 | 0.43-3.52 | 0.70 |
| 18.5-24.9 | 1112 | 1 |  |  |
| 25.0-29.9 | 382 | 1.46 | 0.98-2.20 | 0.07 |
| 30 + | 196 | 2.03 | 1.26-3.25 | 0.003 |
| (missing) | (87) | -- | -- | -- |
| **Mental health history** |  |  |  |  |
| No | 1686 | 1 |  |  |
| Yes | 71 | 1.60 | 0.78-3.30 | 0.20 |
| (missing) | (67) | -- | -- | -- |
| **Total** | **1824** |  |  |  |

Note: Reference groups were selected considering the sample size (the largest sample size) in the subgroups or the most (or the least) risk groups based on expectation.
